# Ciprofloxacin-, Cefazolin-, and Methicilin-Soaked Graphene Paper as an Antibacterial Medium Suppressing Cell Growth

**DOI:** 10.3390/ijms25052684

**Published:** 2024-02-26

**Authors:** Barbara Nasiłowska, Aneta Bombalska, Marta Kutwin, Agata Lange, Sławomir Jaworski, Kamila Narojczyk, Klaudia Olkowicz, Zdzisław Bogdanowicz

**Affiliations:** 1 Institute of Optoelectronics, Military University of Technology, gen. S. Kaliskiego 2, 00-908 Warsaw, Poland; aneta.bombalska@wat.edu.pl (A.B.);; 2 Department of Nanobiotechnology, Institute of Biology, Warsaw University of Life Sciences, Ciszewskiego 8, 02-786 Warsaw, Poland; marta_kutwin@sggw.edu.pl (M.K.); agata_lange1@sggw.edu.pl (A.L.); slawomir_jaworski@sggw.edu.pl (S.J.); 3 Air Force Institute of Technology, Księcia Bolesława 6, 01-494 Warsaw, Poland; klaudia.olkowicz@itwl.pl; 4 Faculty of Mechanical Engineering, Military University of Technology, gen. S. Kaliskiego 2, 00-908 Warsaw, Poland; zdzislaw.bogdanowicz@wat.edu.pl

**Keywords:** graphene oxide, graphene paper, ciprofloxacin, cefazolin, *Staphylococcus aureus*, *Pseudomonas aeruginosa*

## Abstract

This paper presents the results of research on the impact of graphene paper on selected bacterial strains. Graphene oxide, from which graphene paper is made, has mainly bacteriostatic properties. Therefore, the main goal of this research was to determine the possibility of using graphene paper as a carrier of a medicinal substance. Studies of the degree of bacterial inhibition were performed on *Staphylococcus aureus* and *Pseudomonas aeruginosa* strains. Graphene paper was analyzed not only in the state of delivery but also after the incorporation of the antibiotics ciprofloxacin, cefazolin, and methicillin into its structures. In addition, Fourier-Transform Infrared Spectroscopy, contact angle, and microscopic analysis of bacteria on the surface of the examined graphene paper samples were also performed. Studies have shown that graphene paper with built-in ciprofloxacin had a bactericidal effect on the strains of *Staphylococcus aureus* and *Pseudomonas aeruginosa*. In contrast, methicillin, as well as cefazolin, deposited on graphene paper acted mainly locally. Studies have shown that graphene paper can be used as a carrier of selected medicinal substances.

## 1. Introduction

Research on the application possibilities of using graphene and its derivatives in bioengineering is a source of inspiration for many scientists around the world. Graphene oxide (GO) flakes are flat 2D structures [1,2,3]. It is assumed that when >5 layers of carbon atoms are connected to each other there is already another allotropic variant of carbon (graphite and graphite oxide, for example). However, this is not the case with graphene paper (pGO) because graphene oxide flakes, although they are evenly stacked on top of each other, are not connected to each other by chemical bonds. The adhesion of successive GO flakes to each other is related to the process of producing pGO [3].

Publication [4] describes the antibacterial mechanisms of GO interaction, namely, penetration and disruption of the bacterial cell membrane. Elsewhere [5,6,7], descriptions have been presented of the leakage of intracellular contents after penetration and disruption of the cell membrane, as well as oxidative stress through the production of reactive oxygen species and bacterial entrapment (wrapping effect) by GO [8,9].

Kumar et al. [10] presented the results of antimicrobial studies of two water-dispersible graphene derivatives: GO nanoparticles and reduced graphene oxide (rGO). They showed that graphene-based nanomaterials can effectively inhibit the growth of E. coli bacteria while showing minimal cytotoxicity [10].

Ruiz et al. [11] rightly noted that there are many conflicting reports on the biocompatibility and antimicrobial activity of GO. Therefore, they conducted a study to characterize the antimicrobial properties of GO and its biocompatibility with mammalian cells. Studies have shown that adding GO to bacterial culture at a concentration of 25 μg/mL resulted in faster growth of bacteria that achieved higher density than cultures without GO. Scanning electron microscopy also confirmed the presence of dense biofilms in the presence of GO. This study indicated that GO lacks antibacterial, bacteriostatic, and cytotoxic properties in both bacterial and mammalian cells. In addition, GO acted as a general cell growth enhancer by increasing cell adhesion and proliferation [11].

Diverse reports on the antibacterial effect of GO have been verified many times. Most of the research so far leads to the conclusion that GO has more bacteriostatic properties than bactericidal, but this depends on physicochemical properties, particle size, pH, purity, and type of GO [5,6,8,12,13,14,15,16,17,18,19,20,21,22].

To increase the impact of GO derivatives in a controlled and purposeful manner, functionalization is used, meaning the attachment of various functional groups to the structure of GO [23].

One interesting issue is the possibility of using GO as a carrier of a therapeutic substance. GO may have unsaturated bonds, which additionally and after the interaction of plasma can increase in amount. Introducing this into the environment containing the active substance in the form of a drug may cause it to attach to the surface of GO flakes not only physically but also by chemical adsorption [24,25,26]. Matulewicz et al. [23] present the results of the morphology, viability, and proliferation of the T24 and 786-0 cells after subjecting them to GO nanoparticles. They observed that the effects of cytotoxicity are highly dependent on the dose and size of the nanomaterial, so further studies are needed to determine the optimal dose of GO for drug modification [23].

The structure of GO with numerous vacancies makes it resemble a membrane [27,28,29,30]. Thanks to this, GO can gradually release other substances (chemical compounds) that are between the flakes or are attached to its structure [31].

Paper [31] presents the results of studies on the degree of release of ciprofloxacin incorporated into GO and deposited on an orthopedic implant. Studies have shown that GO released ciprofloxacin 5–11 times slower (depending on the solvent used; 5 times in the case of ethanol used and 11 times in the case of water) than in the case of the antibiotic itself deposited on the implant surface.

The gradual release feature of other chemical compounds, such as antibiotics, from the structure of GO allows it to be used locally in the indicated area requiring treatment and not to cover the entire body with antibiotic therapy. This is particularly important in the case of increasing antibiotic resistance [32].

Matulewicz et al. [23] showed that the modification of ciprofloxacin with nanomaterials, such as GO, can increase the cytotoxicity of this chemotherapeutic agent. Ciprofloxacin, often used in clinical practice and taken orally, contains additional compounds, such as HCl and lactic acid additives, which allow for higher solubility and better absorption by the body. In addition, microscopic observations have indicated that ciprofloxacin is released from GO to a much lesser extent, which may result in more effective action.

In the work presented, research was undertaken to supplement the current state of knowledge and the possibility of using pGO as a carrier of therapeutic substances, such as the antibiotics methicillin, cefazolin, and ciprofloxacin.

In addition to the possibility of the gradual release of the antibiotic by GO flakes over a longer period of time (than in the case of the antibiotic alone), the combination of two agents with antibacterial activity is intended to enhance the local effect of inhibiting bacterial growth. An antibiotic deposited on pGO is released more slowly than an antibiotic dissolved only in the medium. In addition, two effects of inducing cell death are combined without the need to use other chemotherapeutic agents to which the bacteria might be resistant.

Complementing the current state of knowledge, we tried to combine two issues by answering the questions of whether pGO used for research has bacteriostatic and bactericidal effects and whether it is possible to use it as a carrier of therapeutic substances.

## 2. Results

### 2.1. Surface Morphology—SEM

**Figure 1:**
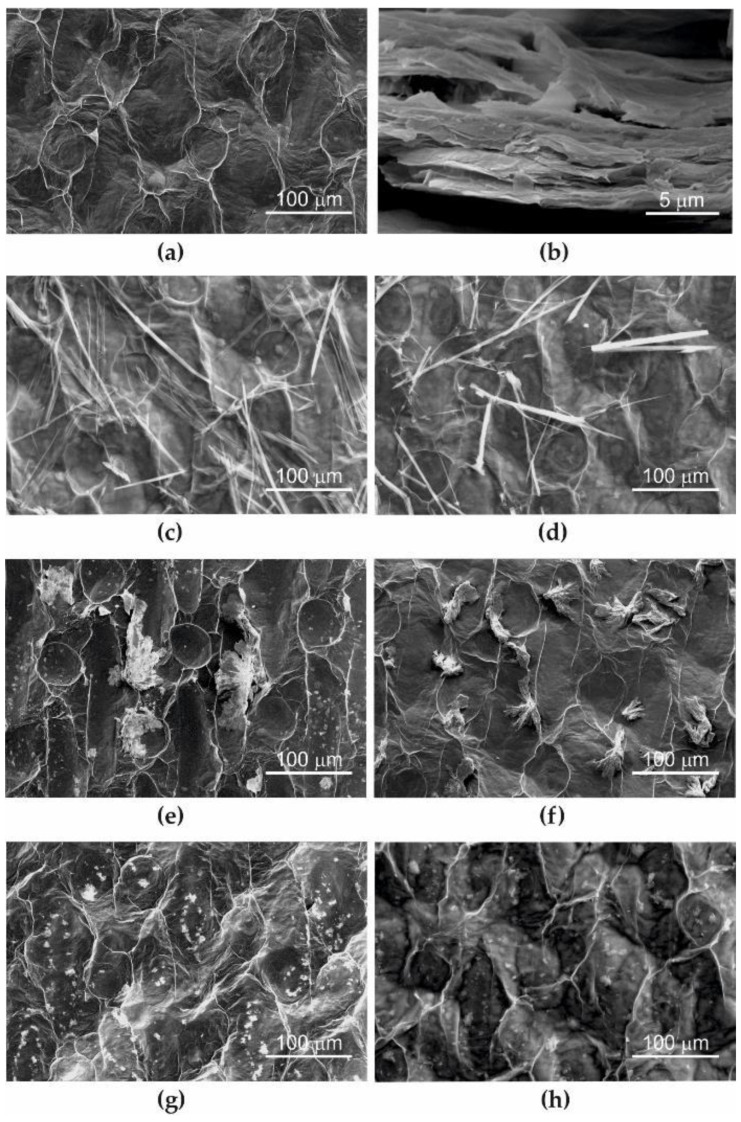
SEM image of pure pGO: (**a**) top view, (**b**) cross-section and graphene paper with tested antibiotics; (**c**) CipW + pGO, (**d**) CipE + pGO, (**e**) CefW + pGO, (**f**) CefDMF + pGO, (**g**) MetW + pGO, (**h**) MetDMF + pGO.

The image of the surface morphology (Figure 1a) of graphene paper (pGO) (not modified in delivery condition) with its cross-section showing numerous GO flakes arranged in parallel (Figure 1b) was taken using a scanning electron microscope. Visible surface irregularities (Figure 1a) are the result of imprinting traces of the piston matrix during pGO production technology [3]. The thickness of pGO was about 150–200 nm.

Figure 1c–h show the surface of pGO after modification with RF plasma (1 min, 100 W) and immersion in solutions with tested antibiotics, i.e., cipofroxacin (CipW + pGO—Figure 1c, CipE + pGO—Figure 1d), cefazoline (CefW + pGO—Figure 1e, CefDMF + pGO—Figure 1f), and methicillin (MetW + pGO Figure 1g, MetDMF + pGO Figure 1h). Sample designations are given in Section 4.1.2.

SEM images (Figure 1c–h) were taken in the central part of the samples on the outer surface of pGO after it had been dried in a vacuum dryer. Clusters of embedded and crystallized antibiotic molecules dissolved according to the manufacturer’s recommendations, and the safety data sheet of the tested substances in water, ethanol, and DMF were observed. The largest concentrations of deposits are visible when water is used as a solvent. This is related to the kinetics of dissolving antibiotics in different solvents [23].

### 2.2. Wettability Tests

**Figure 2:**
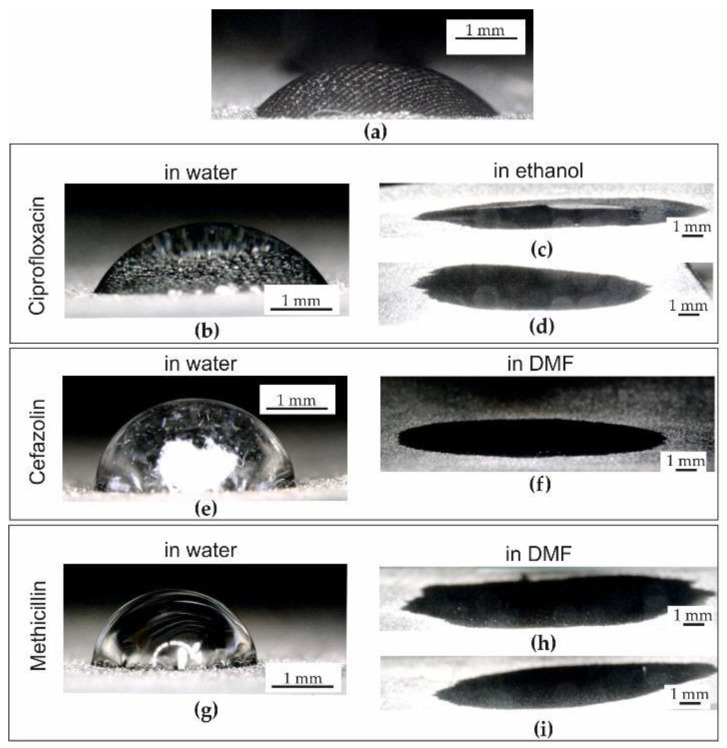
Distilled water (**a**) and solutions of CipW (**b**), CipE (**c**,**d**), CefW (**e**), CefDMF (**f**), MetW (**g**), and MetDMF (**h**,**i**) on pGO during wettability tests.

**Figure 3:**
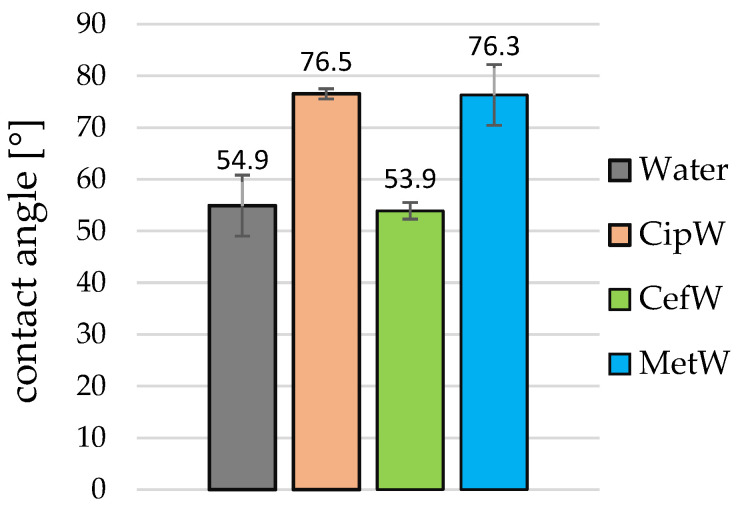
The contact angle values of distilled water and the solutions of CipW, CefW, and MetW on pGO.

The wettability tests of pGO, after applying a drop of distilled water (Figure 2a), and solutions CipW (Figure 2b), CipE (Figure 2c,d), CefW (Figure 2e), CefDMF (Figure 2f), MetW (Figure 2g), and MetDMF (Figure 2h,i) showed that we can determine the contact angle only for aqueous solutions (Figure 2b,e,g) and distilled water itself (Figure 2a). In contrast, antibiotic solutions with ethanol CipE (Figure 2c,d), CefDMF (Figure 2f), and MetDMF (Figure 2h,i) are characterized by complete wettability (0°). Therefore, in Figure 3, only the average results of the wetting angles of distilled water and CipW, CefW, and MetW solutions are given.

It was observed that the largest contact angle close to the contact angle of distilled water (54.9° ± 11.4°) occurred with the CefW solution and was 53.9° ± 1°. In contrast, the mean contact angles of the CipW and MetW solutions were 76.5° ± 5.9° and 76.3° ± 1.6°, respectively.

### 2.3. Surface Chemistry—FTIR

**Figure 4:**
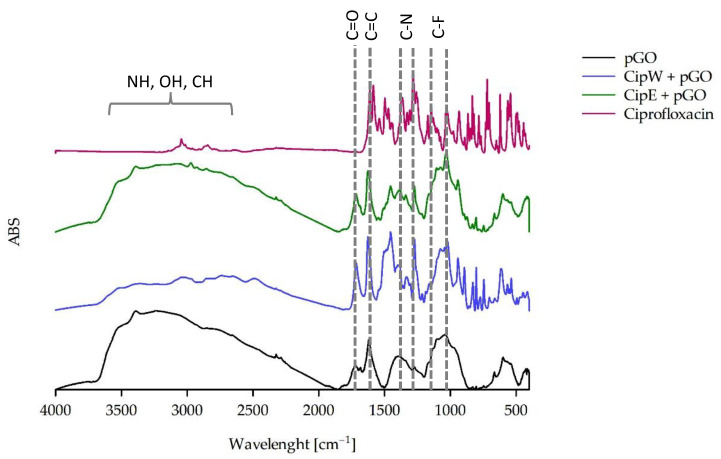
FTIR spectrum of pGO and pGO with adsorbed ciprofloxacin in water (CipW) and ethanol (CipE).

**Figure 5:**
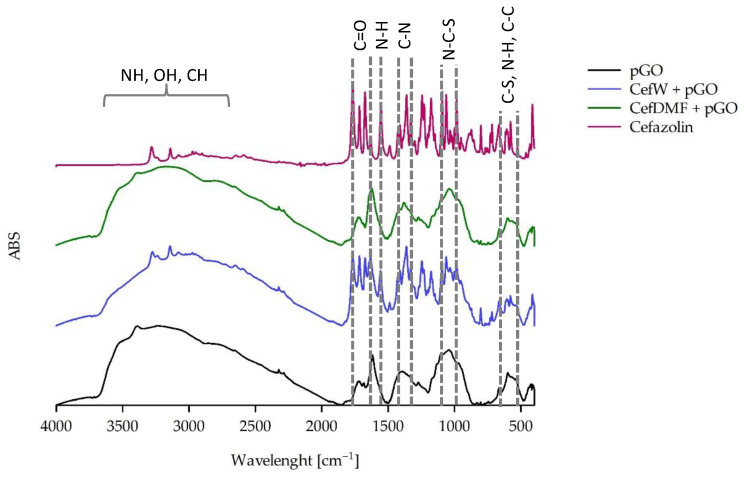
FTIR spectrum of pGO and pGO with adsorbed cefazolin in water (CefW) and DMF (CefDMF).

**Figure 6:**
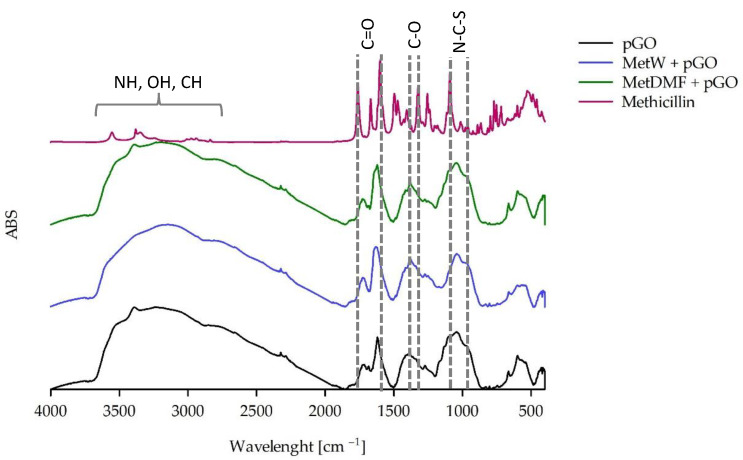
FTIR spectrum of pGO and pGO with methicillin adsorbed in water (MetW) and DMF (MetDMF).

Using FTIR, not only were the spectra of pure pGO and the spectra of antibiotics in the delivery state recorded but the presence of the antibiotics ciprofloxacin (Figure 4), cefazolin (Figure 5), and methicillin (Figure 6) (tested after dissolution according to the recommendations in the safety data sheet Section 4.1.2) on the surface of pGO was also determined.

In the spectra of CipW + pGO and CipE + pGO samples, antibiotic-derived absorption bands of ciprofloxacin were observed (Figure 4). The absorption bands of CipW recorded on the pGO surface were more visible than the CipE bands. These small differences indicate that CipW adsorbed better on the pGO surface. The C=O, C=C, CNC, and C-N [23] bands from ciprofloxacin are more pronounced.

Analysis of the spectra outlines permits the conclusion that cefazolin deposited on pGO from an aqueous solution adsorbed on it, while the presence of the DMF solution on the surface was not observed (Figure 5). Although cefazolin dissolved well in both solvents, the adsorption effect was different and resulted from the properties of the solvents.

This study showed that neither methicillin solution, MetW or MetDMF, was observed on the surface of pure pGO (Figure 6).

### 2.4. Inhibition Tests

The degree of inhibition of bacterial growth was shown as a function of the area not occupied by bacteria around pGO discs. The larger the diameter of this area [cm], the stronger the effect of individual antibiotics.

The antibacterial evaluation study of GO paper was carried out on the basis of the guidelines of [33], which specifies a method for the determination of the effect of antibacterial treatments applied to flat textiles. According to the standard, the indicated incubation time is from 18 to 24 h at a temperature of 37 °C. The incubation time of 24 h was long enough to observe the presence or absence of a growth inhibition zone. A longer period of incubation time could lead to a reduction in the contact of pGO with the agar medium and falsify the final results. This could happen due to an increase in the water content of pGO samples, which can cause a decrease in humidity and inhibit bacterial growth on a solid medium.

**Figure 7:**
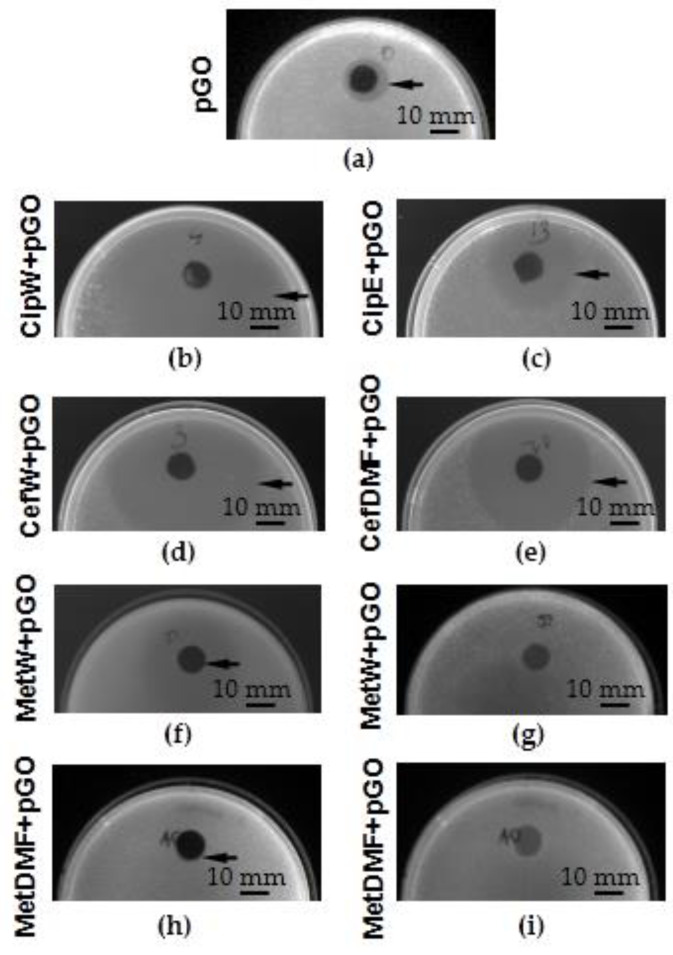
Bacterial growth inhibition zone (indicated by arrows) of *Staphylococcus aureus*; (**a**) pGO, (**b**) CipW + pGO, (**c**) CipE + pGO, (**d**) CefW + pGO, (**e**) CefDMF + pGO, (**f**,**g**) MetW + pGO, (**h**,**i**) MetDMF + pGO.

**Figure 8:**
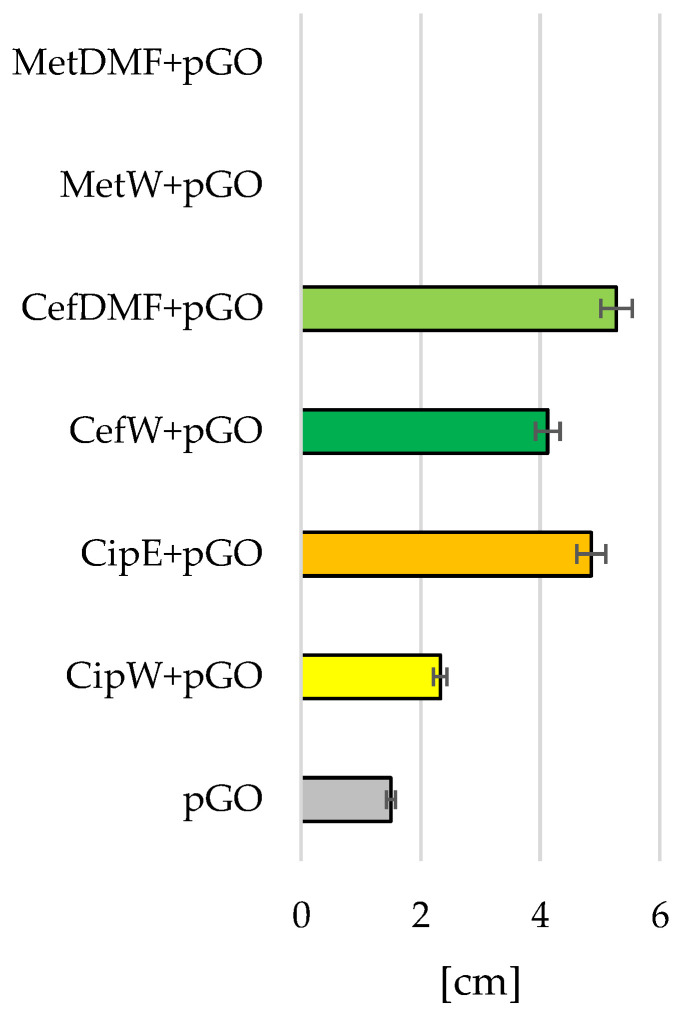
Diameter of the bacterial growth inhibition zone [cm] of *Staphylococcus aureus*.

**Figure 9:**
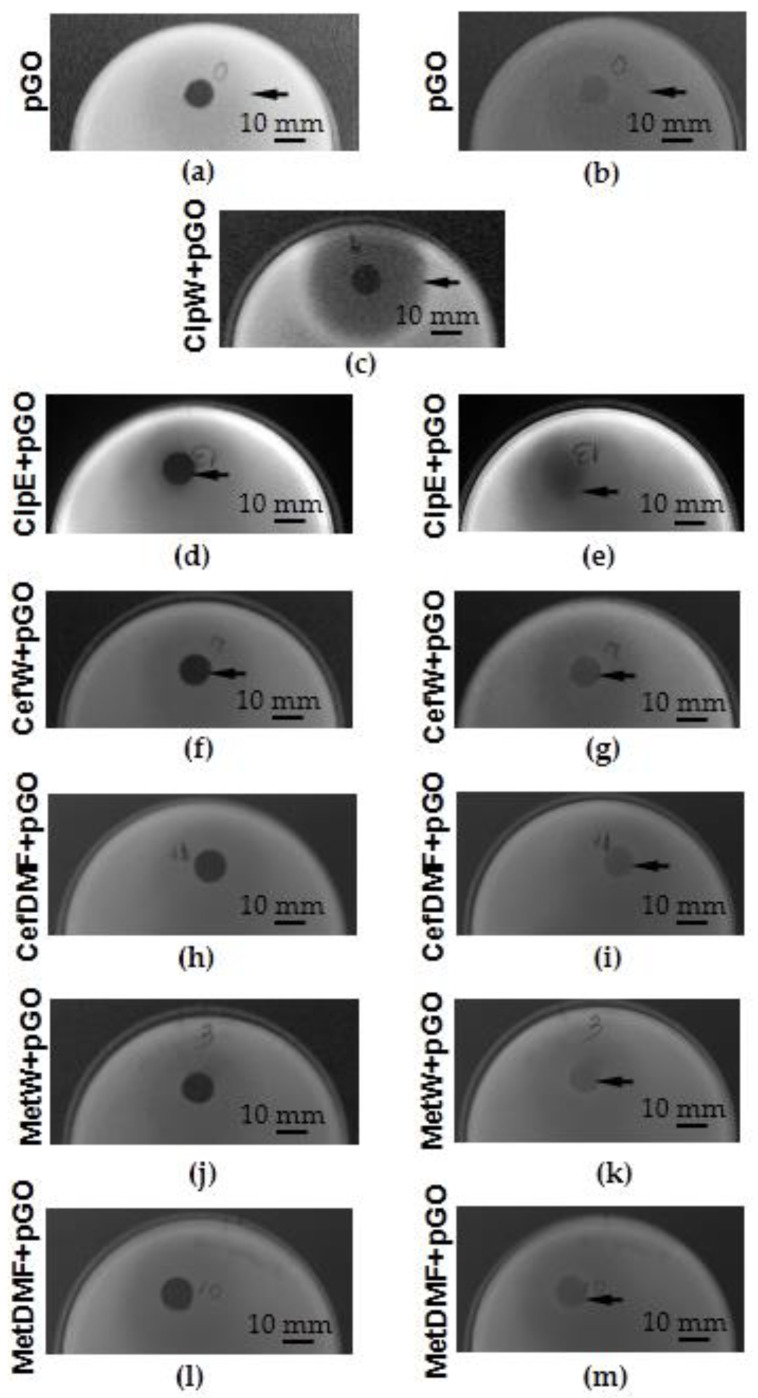
Bacterial growth inhibition zone (indicated by arrows) of *Pseudomonas aeruginosa*; (**a**,**b**) pGO, (**c**) CipW + pGO, (**d**,**e**) CipE + pGO, (**f**,**g**) CefW + pGO, (**h**,**i**) CefDMF + pGO, (**j**,**k**) MetW + pGO, (**l**,**m**) MetDMF + pGO.

**Figure 10:**
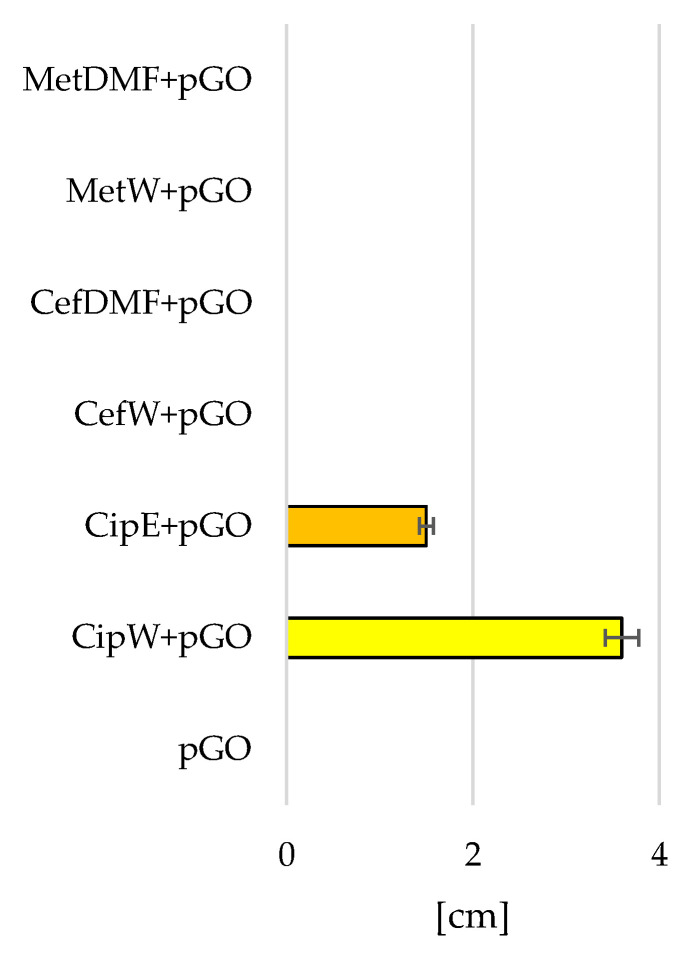
Diameter of the bacterial growth inhibition zone [cm] of *Pseudomonas aeruginosa*.

The analysis of the results of the degree of growth inhibition of *Staphylococcus aureus* (Figure 7 and Figure 8) and *Pseudomonas aeruginosa* bacteria (Figure 9 and Figure 10) showed that the most effective interaction was with the solution of the antibiotic ciprofloxacin CipW + pGO and CipE + pGO deposited on pGO (Figure 7b,c and Figure 9c).

The graphene paper (pGO) examined showed bactericidal activity on strains of *Staphylococcus aureus* bacteria (Figure 7a and Figure 8) (1.5 cm) and locally on *Pseudomonas aeruginosa* (Figure 9a,b and Figure 10). Similar in nature were CefW + pGO and CefDMF + pGO samples, whose zone of bacterial growth inhibition on *Staphylococcus aureus* was 2.32 cm and 5.27 cm, respectively, and on *Pseudomonas aeruginosa*, and the impact was mainly local.

In contrast, methicillin deposited on pGO acted only locally for both bacterial strains, without causing a degree of bacterial inhibition.

**Figure 11:**
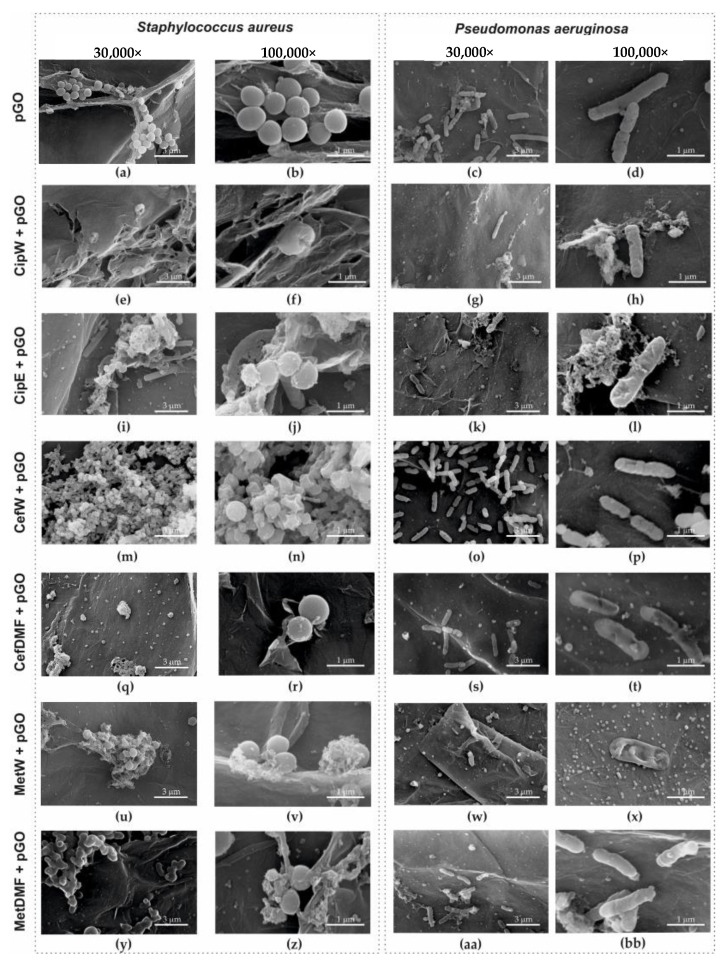
Morphology of the cells *Staphylococcus aureus* on the surface of (**a**,**b**) pGO, (**e**,**f**) CipW + pGO, (**i**,**j**) CipE + pGO, (**m**,**n**) CefW + pGO, (**q**,**r**) CefDMF + pGO, (**u**,**v**) MetW + pGO, (**y**,**z**) MetDMF + pGO, magnification 30,000× (**a**,**e**,**i**,**m**,**q**,**u**,**y**), 100,000× (**b**,**f**,**j**,**n**,**r**,**v**,**z**), and *Pseudomonas aeruginosa* on the surface of (**c**,**d**) pGO, (**g**,**h**) CipW + pGO, (**k**,**l**) CipE + pGO, (**o**,**p**) CefW + pGO, (**s**,**t**) CefDMF + pGO, (**w**,**x**) MetW + pGO, (**aa**,**bb**) MetDMF + pGO, magnification 30,000× (**c**,**g**,**k**,**o**,**s**,**w**,**aa**), 100,000× (**d**,**h**,**l**,**p**,**t**,**x**,**bb**).

Using scanning electron microscopy, the fixed zone of growth inhibition (Section 4.2.2 and Section 4.2.3) of *Staphylococcus aureus* and *Pseudomonas aeruginosa* (Figure 11) is shown after 24 h for the following samples:

- pGO (*S. aureus* Figure 11a,b and *P. aeruginosa* Figure 11c,d);
- CipW + pGO (*S. aureus* Figure 11e,f and *P. aeruginosa* Figure 11g,h);
- CipE + pGO (*S. aureus* Figure 11i,j and *P. aeruginosa* Figure 11k,l);
- CefW + pGO (*S. aureus* Figure 11m,n and *P. aeruginosa* Figure 11o,p);
- CefDMF + pGO (*S. aureus* Figure 11q,r and *P. aeruginosa* Figure 11s,t);
- MetW + pGO (*S. aureus* Figure 11u,v and *P. aeruginosa* Figure 11w,x);
- MetDMF + pGO (*S. aureus* Figure 11y,z and *P. aeruginosa* Figure 11aa,bb).

To illustrate the various sample fragments, images (Figure 11) were presented for magnifications of 30,000× (*S. aureus*, Figure 11a,e,i,m,q,u,y, and *P. aeruginosa*, Figure 11c,g,k,o,s,w,aa) and 100,000× (*S. aureus*, Figure 11b,f,j,n,r,v,z, and *P. aeruginosa*, Figure 11d,h,l,p,t,x,bb).

On the surface of pGO (Figure 11a–d), no numerous clusters of bacteria were observed. However, the number was much higher than in the case of CipW + pGO (Figure 11e–h), CipE + pGO (Figure 11i–l), CefW + pGO (Figure 11m–p), CefDMF + pGO (Figure 11q–t), MetW + pGO (Figure 11u–x), and MetDMF + pGO (Figure 11y,z,aa,bb).

It was also observed that the morphology of the outer layers of Staphylococcus aureus and Pseudomonas aeruginosa bacteria located on CefW + pGO samples (Figure 11m–p) is not uniform. Such morphological changes may be the result of bacterial cell death due to perforation of the outer membrane, resulting in reduced cell proliferation and bacterial immobilization on the surface of CefW + pGO samples. The number of bacterial clusters on CefW + pGO samples (Figure 11m–p) is similar to pGO samples (Figure 11a–d). However, the cells have a disturbed morphology of the outer membrane, indicating degradation processes.

It is also interesting that methicillin acts locally without causing a degree of inhibition of bacterial growth. On the surface, however, the condition of the bacterial cell membrane is disturbed by MetW + pGO (Figure 11x), and the number of MetW + pGO (Figure 11u–x) and MetDMF + pGO (Figure 11y,z,aa,bb) is negligible.

## 3. Discussion

Wettability tests were performed to investigate the distribution of antibiotic solutions on the surface of pGO.

Graphene paper (pGO) and the antibiotic solutions ciprofloxacin (CipW, CipE), cefazolin (CefW, CefDMF), and methicillin (MetW, MetDMF), together with appropriate solvents (distilled water, ethanol, and DMF), exhibit hydrophilic properties. However, wettability studies have shown significant differences in the effect of these solvents on pGO. Antibiotics dissolved in water interacted with pGO in a hydrophilic manner (θ = 53°, 79°, and 76° CefW, CipW, MetW, respectively).

The higher wettability of pGO when in contact with an antibiotic allows the antibiotic to penetrate deep into the structures of GO flakes arranged in parallel. As a result, a longer release rate of the antibiotic is observed, which is thus more effective on bacterial biofilm. This is particularly seen in the *Staphylococcus aureus* strain. However, this relationship is less evident in *Pseudomonas aeruginosa* (Figure 10), which may be due to higher concentrations of ciprofloxacin dissolved in water than are used in the case of ethanol.

Dissolution of antibiotics in ethanol, in the case of ciprofloxacin, and DMF, in the case of cefazolin and methicillin, cause a situation where they also behave in a hydrophilic manner when in contact with pGO. However, the contact angle θ cannot be measured because it is 0.

Experiments using FTIR-ATR showed the different abilities of antibiotics to exhibit adsorption on the surface of pGO. The absence of adsorption was shown by antibiotics dissolved in DMF. Their presence was recorded for aqueous solutions, with the exception of methicillin, which was not registered for any solvent variant. The presence of antibiotics recorded by the ATR method is largely consistent with the results of antibacterial activity. ATR-positive CipW + pGO, CipE + pGO, and CefW+ pGO samples also showed antibacterial activity against *Staphylococcus aureus* and *Pseudomonas aeruginosa*. In the case of methicillin, no presence on the surface of pGO was recorded, nor was antibacterial activity observed.

Figure 5 presents cefazolin’s ATR spectra on the surface of pGO. Its presence was not recorded when it was dissolved in DMF (CefDMF + pGO), but it was shown to have antibacterial activity for *S. aureus*. It is difficult to explain this case, especially since the effect of this sample is stronger than for pGO itself, so it cannot be a matter of lack of adsorption (in which case the lack of the ATR spectrum remains unknown).

Studies of the growth inhibition zone of *Staphylococcus aureus* and *Pseudomonas aeruginosa* on samples made of pGO showed that it has a local bactericidal effect. Ciprofloxacin belongs to antibiotics from the quinolone group. The action mechanism of this group of antibiotics is based on the inhibition of the replication of genetic material (bacterial DNA), leading to the death of the bacterial cell. Ciprofloxacin is involved in the inhibition of topoisomerase activity (DNA gyrase, and topoisomerase IV). The antibiotics cefazolin and methicillin used in this study belong to the group of beta-lactam antibiotics. They use a mechanism consisting of inhibiting the synthesis of the bacterial cell wall, comprising mainly peptidoglycan. Disruption of cell wall synthesis causes the bacterial cell to lose stability in the environment and undergo lysis. Bacterial enzymes with autolysin activity are involved in this process [34].

*Staphylococcus aureus* and *Pseudomonas aeruginosa* are becoming the most common pathogens in hospitals. These two microbiomes differ in microscopic structure and the activity of genes responsible for antibiotic resistance [35]. They have different responses to antibiotics such as ciprofloxacin, cephalosporin, and methicillin.

Graphene paper (pGO) soaked in ciprofloxacin caused a significant increase in the diameter of the zone of bacterial growth inhibition in relation to the pGO control group (Figure 8 and Figure 10). Differences in the diameter of the growth inhibition zone between bacterial strains could result from the difference in the proliferation rate of the bacterial cells tested. An observation of changes in bacterial cell morphology (Figure 11) showed that pGO soaked in ciprofloxacin not only caused damage to the outer layers of bacterial cells but also reduced the density of bacterial cells observed on pGO and caused an increase in bacterial immobilization in relation to the control group.

The results obtained indicate a bifunctional mechanism of action of pGO with embedded antibiotics belonging to the β-lactam group, which is to say, methicillin and cefazolin. Methicillin acts by specifically inhibiting cross-linkage between the linear peptidoglycan polymer chains that make up a major component of the cell wall of gram-positive bacteria [36]. On the other hand, cefazolin is a first-generation use of cephalosporin for IV administration. It is mostly used against gram-positive pathogens but also shows limited activity against gram-negative bacteria [37].

Functionalization of pGO with antibiotics β-lactam led to damage to the outer membranes of gram-negative bacterial cells—*Pseudomonas aeruginosa*—and caused lethal damage to gram-positive bacterial cells—*Staphylococus aureus* (Figure 11n). Graphene paper (pGO) as a carrier for cefazolin and methicillin caused mechanical damage to the outer membranes of both bacterial strains, increasing the activity of antibiotics. Moreover, in the image of bacterial cell morphology of *Staphylococus aureus* and *Pseudomonas aeruginosa* (Figure 11), immobilization of bacterial cells on pGO was observed, limiting the possibility of metabolic activity of bacterial strains.

The antibacterial mechanism of pGO is related to its bidirectional effects on bacteria, including its physical and chemical activity. The physical interaction of pGO with bacterial strains is most often the result of physical damage to the outer membranes of bacteria by the sharp edges of graphene. Chemically, graphene paper, like other graphene materials, can lead to oxidative stress generated by charge transfer and the formation of reactive oxygen species (ROSs) [38]. In our study, the antibacterial activity of pGO resulted from a combination of the mechanisms of action of selected antibiotics and the physicochemical properties of graphene itself, limiting bacterial growth.

Samples of pGO together with deposited antibiotics had a bactericidal effect on all tested bacterial strains. However, the largest zone of bacterial growth inhibition was observed for samples with embedded ciprofloxacin (Cip W + pGO, CipE + pGO), while the weakest results were for samples using methicillin (MetW + pGO, MetDMF + pGO).

## 4. Materials and Methods

This paper presents structural and bacterial studies of the use of the therapeutic substances ciprofloxacin, methicillin, and cefazolin deposited on pGO. Structural studies were performed using a scanning electron microscope, FTIR spectroscopy, and contact angle, while bacteriological studies were performed on strains of *Staphylococcus aureus* (ATCC 25923) and *Pseudomonas aeruginosa* (ATCC 27853).

In the first stage, antibiotic solutions were prepared according to the recommendations of product safety data sheets (Merck Life Science Ltd., Poznań, Poland). Subsequently, pGO was activated using a 1 min 100 W plasma device (prep III device, Garfield Ave, Westchester, PA, USA). Immediately after removal from the plasma chamber, the samples were immersed for 20 s in a beaker containing a dissolved antibiotic. In the last stage, the samples were dried.

### 4.1. Materials

#### 4.1.1. Graphene Paper

Graphene paper (pGO) was purchased from the Department of Chemical Synthesis and Flake Graphene, Łukasiewicz Research Network, Institute of Electronic Materials Technology (IEMT, Warsaw, Poland). Its characteristics, including structural studies, are described in detail in the Supplementary Materials in reference [3]. 

Graphene paper was cut in the form of discs with a diameter of 10 mm using a pipe punch.

#### 4.1.2. Antibiotics

Three therapeutic substances were tested, which were the following antibiotics:

- Ciprofloxacin (Merck Life Science, Cat. No: 17850, Sigma-Aldrich, Poznań, Poland);
- Cefazolin (Merck Life Science, Cat. No: PHR129, Sigma-Aldrich, Poznań, Poland);
- Methicillin, sodium salt (Merck Life Science, Cat. No: 51454, Sigma-Aldrich, Poznań, Poland).

The following samples were prepared:

- pGO—graphene paper CipW + pGO—graphene paper immersed in ciprofloxacin dissolved according to the safety data sheet in water (35 g ciprofloxacin in 1 mL of water, 105 M/L);
- CipE + pGO—graphene paper immersed in ciprofloxacin dissolved according to the safety data sheet in ethanol (1.6 g ciprofloxacin in 1 mL of ethanol, 4.82 M/L);
- CefW + pGO—graphene paper immersed in cefazolin dissolved according to the safety data sheet in water (20 g cefazolin in 1 mL of water, 44 M/L);
- CefDMF + pGO—graphene paper immersed in cefazolin dissolved according to the safety data sheet in DMF (10 g cefazolin in 1 mL of DMF, 22 M/L);
- MetW + pGO—graphene paper immersed in methicillin dissolved according to the safety data sheet in water (10 g methicillin in 2 mL of water, 13 M/L);
- MetDMF + pGO—graphene paper immersed in methicillin dissolved according to the safety data sheet in DMF (20 g methicillin in 20 mL of DMF, 2.62 M/L).

Different solvents had to be used since the antibiotics dissolve at different rates.

### 4.2. Methods

#### 4.2.1. Surface Morphology

- Scanning Electron Microscope—SEM

The surface morphology of pGO was determined using scanning electron microscopy (SEM) (Quanta 250 FEG SEM, FEI, Hillsboro, OR, USA). An SEM image was created with a distributed detector (ETD-BSE, FEI, Hillsboro, OR, USA) with an acceleration voltage of 5 kV for GO and 10 kV. For each sample, 10–20 images were taken. The tests were carried out on the surface near the center of the cut disc with a diameter of 5 mm.

- An FTIR (Fourier-Transform Infrared Spectroscopy) study of the surface chemical composition was conducted.

Pure pGO soaked in antibiotics in various solvents was analyzed by FTIR (Nicolet IS50, FTIR, ThermoFisher SCIENTIFIC, Waltham, MA, USA) to determine the presence or absence of antibiotics adsorbed on its surface. Samples were measured on both sides using ATR (total internal reflection) in a range of 400–4000 cm^−1^ with a resolution of 4 cm^−1^ and 64 scans. Each side of pGO was measured 4 times.

#### 4.2.2. Wettability Tests

The wetting angle of pGO was measured using an optical microscope (6000 VHX, Keyence Corporation, Osaka, Japan). Distilled water and the following solutions were used in the wettability studies: CipW (ciprofloxacine dissolved in water), CipE (ciprofloxacin dissolved in ethanol), CefW (cefazolin dissolved in water), CefDMF (cefazolin dissolved in DMF), MetW (methicillin dissolved in water), and MetDMF (methycelin dissolved in DMF).

Solutions of antibiotics (CipE, CipW, CefW, CefDMF, MetW, MetDMF) and water were collected using a syringe dispenser. Then, drops of a suitable solution with a volume of 3 µL from a constant height of 5 mm were lowered onto the surface of pGO, and a picture was taken. The contact angle was determined on the basis of the photograph and the microscope software results. For each solution, 5 measurements were made, based on which the mean and standard deviation were calculated.

#### 4.2.3. Bacteriological Experiments

*Staphylococcus aureus* (ATCC 25923) and *Pseudomonas aeruginosa* (ATCC 27853) were obtained from the American Type Culture Collection (ATCC) in the form of spore suspension, and bacterial strains were maintained in 20% (*v*/*v*) glycerol at −20 °C. Before use in experiments, glycerol was removed by washing with distilled water. Bacterial strains were cultured in tryptic soy agar (TSA) in standard conditions (24 h, 37 °C).

A total of 10 mL of nutrient agar (BioMaxima, Lublin, Poland) was placed on Petri dishes (90 mm in diameter). Then, 5 mL of nutrient agar inoculated with appropriate bacterial suspension (1.5 × 10^8^ cells/mL) was poured on the previously prepared Petri dishes. Paper discs (for *S. aureus* and *P. aeruginosa*) were placed onto solidified agar, and plates were incubated for 24 h at 37 °C. Results were determined by the zone of growth inhibition.

The bacterial strains used in this study result from the recommendations in the [33], which specifies a method for the determination of the effect of antibacterial treatments applied to flat textiles. These were selected on the basis of a literature review, which indicated that *Staphylococcus aureus* and *Pseudomonas aeruginosa* infections are among the most common etiogenic agents in nosocomial infections [39].

#### 4.2.4. Imaging of Bacterial Cells

A scanning electron microscopy (SEM) analysis of bacterial samples was performed by means of an FEI Quanta 200 electron microscope (FEI Co., Hillsboro, OR, USA). The bacteria samples were rinsed in PBS (0.01 M, pH 7.2; P4417, Sigma-Aldrich, Poznań, Poland) and then fixed in 2.5% glutaraldehyde (G5882, Sigma) for 1 h, washed twice with 0.1 M PBS (0.01 M, pH 7.2; P4417, Sigma), and placed on aluminum SEM stubs. The SEM stubs were kept in a moist atmosphere for 1 h, washed with PBS (0.01 M, pH 7.2; P4417, Sigma), postfixed in 1% osmium tetroxide (75632, Sigma) for 1 h, rinsed in distilled water, and dehydrated in graded ethanol. After critical point drying with liquid CO_2_ in a vacuum apparatus (Polaron CPD 7501, Quorum Technologies, Newhaven, East Sussex, UK) and coating with a 5.12 nm gold layer gold using an EM ACE 600 high-vacuum sputterer (Leica Microsystems, Wetzlar, Ger-many), the samples were inspected by SEM at 5 KeV (Quanta 250 FEG SEM, FEI, Hillsboro, OR, USA).

## 5. Conclusions

The analysis of the research results allowed the following conclusions to be formed:

- Graphene paper had a flake structure consisting of numerous layers of GO adjacent to each other;
- Wettability studies showed that antibiotics dissolved in distilled water had a smaller contact angle than other solvents, such as ethanol and DMF. The smallest wetting angle (0) was characterized by solutions of antibiotics dissolved in ethanol and DMF, i.e., CipE, CefDMF, and MetDMF. In contrast, the largest wetting angle occurred in the case of the CipW solution, similar to the wetting angle of distilled water;
- In contrast to pGO samples and pGO containing ciprofloxacin (CipW, CipE) and cefazolin (CefW, CefDMF), no methicillin peaks derived from the spectrum (FTIR) were observed on the surface of the graphene paper (MetW, MetDMF);
- Studies of bacterial growth inhibition and the condition of fixed bacteria on the surface of pGO showed that the greatest bactericidal impact was characterized by CipW and CipE samples;
- The graphene paper used for the research was characterized by a local bacteriostatic/bactericidal effect and showed potential possibilities for use as a drug carrier to inhibit bacterial growth.

## Data Availability

Data are contained within the article.
